# High Antioxidant Capacity of *Lacticaseibacillus paracasei* TDM-2 and *Pediococcus pentosaceus* TCM-3 from Qinghai Tibetan Plateau and Their Function towards Gut Modulation

**DOI:** 10.3390/foods12091814

**Published:** 2023-04-27

**Authors:** Tingyu Yang, Xueni Fan, Diantong Li, Tingting Zhao, Dan Wu, Zhenjiang Liu, Danfeng Long, Bin Li, Xiaodan Huang

**Affiliations:** 1 School of Public Health, Lanzhou University, Lanzhou 730033, China; yangty20@lzu.edu.cn (T.Y.);; 2 Institute of Animal Husbandry and Veterinary, Tibet Academy of Agricultural and Animal Husbandry Sciences, Key Laboratory of Animal Genetics and Breeding on Tibetan Plateau, Ministry of Agriculture and Rural Affairs, Lhasa 850000, China; 3 National Engineering Laboratory for AIDS Vaccine, School of Life Sciences, Jilin University, Changchun 130012, China

**Keywords:** lactic acid bacteria, Qinghai-Tibetan plateau, antioxidant capacity, gut microbiota

## Abstract

Probiotic supplementation is a key therapeutic strategy for promoting gut health and maintaining gut homeostasis by modulating functional microbiota. In this study, we isolated two lactic acid bacteria (LAB) strains, *Pediococcus pentosaceus* TCM-3 and *Lacticaseibacillus paracasei* TDM-2, from Qinghai-Tibetan plateau, and evaluated their probiotic properties and antioxidant bioactivity. In which, TDM-2 had higher T-AOC activity than either TCM-3 or LGG (4.10 μmol/mL vs. 3.68 and 3.53 μmol/mL, respectively, *p* < 0.05). These strains have shown high antioxidant activity compared to the LAB strains and were found to be acid and bile salt tolerant, confronting the safety issues of antibiotic resistance and the capability of surviving in simulated gastric and intestinal juices. In vitro fermentation experiments with human gut microbiota revealed significant differences in microbial community composition between samples supplemented with TCM-3 and TDM-2 and those without. The addition of these two strains resulted in an enrichment of beneficial taxa, such as the *Pediococcus*, *Lactobacillus*, and *Clostridium_sensu_strictos* at the genus level, and Firmicutes and Proteobacteria at the phylum level. Notably, the TCM-3 group exhibited higher short-chain fatty acid production than the TDM-2 group and untreated controls (acetic acid at 12 h: 4.54 mmol L^−1^ vs. 4.06 mmol L^−1^ and 4.00 mmol L^−1^; acetic acid at 24 h: 4.99 mmol L^−1^ vs. 4.90 mmol L^−1^ and 4.82 mmol L^−1^, *p* < 0.05). These findings demonstrate that LAB supplementation with high antioxidant capacity and probiotic properties can promote gut health by modulating functional microbiota and is enriching for beneficial taxa. Our study provides guidance for therapeutic strategies that use novel LAB strains to maintain gut homeostasis and functional microbiota modulation.

## 1. Introduction

Gut microbiota have been proposed to function in concert, similar to an organ, in contributing to human health, supporting the maintenance of metabolic function, and protecting against illness [1]. Furthermore, gut microbiota is significantly influenced by host and diet. In our previous study [2], metagenome sequencing indicated that gut microbiota can substantially differ among people living in close regional proximity in a diet-driven manner. Moreover, many studies have investigated the applications of probiotics, prebiotics, and post-biotics to regulate gut microbiota. For example, the findings of several studies support that probiotic lactic acid bacteria (LAB), such as *Lactobacillus*, *Streptococcus*, and *Enterococcus,* can facilitate the establishment of gut microbial homeostasis [3,4], enhancing intestinal barrier function [5], alleviating diarrhea [6], maintaining a stable set of core indigenous microbiota [7], and preventing gastrointestinal infections [7,8]. Other recent work has suggested that probiotic strains can potentially enhance immune responses and positively influence microbial homeostasis in the gastrointestinal tract [9].

LAB have thus been established as one of the essential bacterial groups contributing to the health of humans, with members reported to show a range of biological effects, including antioxidant [10,11,12,13], anti-inflammatory [14,15], antibacterial [15], and cholesterol-lowering activities, among others [16,17]. LAB strains are used widely in food products around the globe and are cultured as probiotics to potentially prevent or treat a wide variety of human diseases and disorders, such as metabolic syndromes, chronic disease, and neurological or neurodegenerative diseases [18,19,20], many of which (e.g., diabetes, cancer, and Alzheimer’s disease) are intimately linked with oxidative stress caused by excess intracellular reactive oxygen species (ROS) production [21,22]. Furthermore, increasing experimental evidence supports the possibility that the potentially beneficial antioxidative effects of LAB can be harnessed to treat, or even prevent, some chronic diseases [8,23,24,25]. Although the antioxidant properties of probiotic LAB and their function in the modulation gut microbiota have been demonstrated both in vitro and in vivo, the mechanism behind them are not fully understood [26].

Among the various effects of these probiotic strains, antioxidant activity may provide some of the strongest effects to potentially improve host health. Tsao et al. [27] reported that supplementing the diet with *L. salivarius* AP-32 can enhance host antioxidant enzyme activity via direct and indirect modes, such as by altering the composition of fecal microbiota to specifically enrich beneficial commensal taxa in Parkinson’s disease model rats. Antioxidant potential was also proposed to serve as a possible biomarker for probiotic LAB function in the human gut, as reviewed by Mei et al. [28]. Therefore, screening novel LAB, especially from extreme environments where increased antioxidant effects may be a determining factor in adaptation and survival, is a topic of considerable research interest to facilitate the discovery and application of effective probiotic strains.

Spanning 2.5 million km^2^ with an average altitude of 4000 m above sea level [29], the Qinghai-Tibetan plateau is rich with distinctive bioresources, including rare animals [30,31,32,33,34], highland flora [35,36], and functional microorganisms, despite the low oxygen levels and prolonged extreme cold [37,38,39]. Recent research efforts have focused on isolating and characterizing gut microbes that might contribute to host adaptation to environmental stresses. For instance, *Lactobacillus plantarum* isolation from Tibetan kefir grains was reported to exhibit potent antioxidant activity and cholesterol-lowering effects in mice/humans/in vitro [11]. Recent work by Navani et al. [40] revealed that asparaginase generated by *Lactobacillus brevis* in the Himalayan yak cheese, Chhurpi, could inhibit the growth and proliferation of cancer cells. Collectively, these studies illustrated the strong potential for identifying functional microbes in this extreme environment, especially lactic acid bacteria (LAB).

In this study, two LAB strains were identified and isolated from the dairy products of Qinghai-Tibetan plateau velvet cows, which exhibited high antioxidant capacity in vitro. We then used metagenomic analysis and gas chromatography-mass spectrometry (GC-MS) to investigate their impacts in modulating microbial composition and diversity, as well as microbial short-chain fatty acid (SCFA) production. Findings in this study depict the significant antioxidant activities of these LAB strains from the Qinghai-Tibetan plateau, supporting their further application in functional food production and potential development as probiotics for treating gastrointestinal dysbiosis.

## 2. Materials and Methods

### 2.1. Isolation of LAB

Strains isolated from fresh milk samples of Jersey cattle and Yak were collected from diverse locations on the Qinghai-Tibetan plateau (31°58′15.60″ N, 88°47′31.20″ E). The samples underwent serial dilution with DeMan, Rogosa, and Sharpe Broth (MRS, pH 5.7 ± 0.2) up to 10^−1^–10^−9^. They were then inoculated onto M17 agar (pH 7.2 ± 0.2) and Man Rogosa Sharpe (MRS) agar (pH 5.7 ± 0.2) and incubated at 37 °C for 48 h. After the incubating period, potential lactic acid bacteria (LAB) were accessed with a sterile needle and inoculated into MRS broth, which was then incubated at the temperature of 37 °C for 8 h. All the samples were purified further by via streak plating and preliminary identification was performed based on their morphological and staining characteristics, following the protocol outlined by Kozaki et al. [41]. Gram-positive purified cultures were then inoculated into MRS broth pipes, which were supplemented with sterile glycerol (25 mL/100 mL) and stored at −80 °C for the subsequent analysis.

### 2.2. Identification of LAB

The previously described Adimpong et al. [42] modified method was followed to identify LAB. The total genomic DNA was obtained from the LAB using the Solarbio Genomic DNA extraction kit for Gram-positive bacteria, following the manufacturer’s instructions. Amplification of the complete sequence of the 16S rRNA gene was conducted by PCR, using forward primer 27F (5′-AGA GTT TGA TCC TGG CTC AG-3′) and reverse primer 1492R (5′-AAG GAG GTG ATC CAG CCG CA-3′). The PCR reaction mixture (50 μL) contained 24 μL of GoTaq Hot Start Colorless Master Mix, 1.5 μL of each primer, 2 μL of 25 mM MgCl_2_, 19 μL of UltraPure Distilled Water, and 2 μL of bacterial DNA. The PCR was executed in a Thermal Cycler with the following conditions: the initial pre-denaturing step was conducted at 95 °C for 2 min, which was followed by 33 cycles at 95 °C (30 s), 51 °C (30 s), and 72 °C (30 s), and a further final extended period of 2 min at 72 °C. The amplified DNA was analyzed by electrophoresis on a 1% (*w*/*v*) agarose gel (Sangon Biotech, Shanghai, China) containing 50 mL 1×TBE buffer and a DNA marker (Sangon Biotech, 1500 bp) at 100 V for 30 min, and it can be visualized on the electrophoresis apparatus.

The PCR amplification was executed with the PTC-100 Thermal Cycler. The generated PCR products were analyzed on a 1% (*w*/*v*) agarose gel (Sangon biotech, Shanghai, China) after staining with ethidium.

Purification and sequencing of PCR products by the *P. pentosaceus* TCM-3 and *L. paracasei* TDM-2 Sequence Assembly Program, and the resulting sequences, were analyzed with the BioEdit Sequence Alignment Editor 7.1.11 software (Raleigh, North Carolina, USA.) and Chromas 2.6.5 (Brisbane, Queensland, Australia). The consensus sequences were compared to the GenBank DNA database using the BLAST algorithm and RDP to identify the closest known relative taxa based on the 16S rRNA gene homology [43]. The identification was performed using the software MEGA 6.0 (Tempe, Arizona, USA.) and based on the highest similarity for the closest related species and the 99–100% homology. The sequences of the 16S rRNA genes of the separates were submitted in GenBank with accession numbers: OQ449696, OQ449697.

### 2.3. Antioxidant Activity Screening of Probiotics

#### 2.3.1. Preparation of Cell-Free Extracts

In this study, the cell-free extracts were prepared using a modified method by Lin et al. [44]. Specifically, the bacterial specimens were activated three times, the precipitation was collected via centrifugation. After washed three times with phosphate-buffered saline (PBS), they were finally resuspended to a concentration of 1 × 10^8^ colony forming units per milliliter (CFU mL^−1^). Next, the bacterial cells were broken down through sonication in an ice bath, which consisted of five-second bursts, followed by five-second pauses, and a final sonication period of 30 min at 80% power. The supernatant was subsequently collected as a cell-free extract via centrifugation at 12,000 rotations per minute for 10 min at 4 °C.

#### 2.3.2. H_2_O_2_-Resistant Ability

The hydrogen peroxide (H_2_O_2_) resistant ability of lactic acid bacteria (LAB) strains was assessed using a modified method from Leite et al. and Xu et al. [45,46]. Each strain was individually injected into MRS broth containing different concentrations of H_2_O_2_ (0 mmol L^−1^, 0.5 mmol L^−1^, 1 mmol L^−1^, and 2.0 mmol L^−1^) and was incubated anaerobically for 48 h at 37 °C. The cultures that demonstrated higher growth under H_2_O_2_ stress were selected for further analysis.

#### 2.3.3. DPPH Radical Scavenging Activity

To evaluate the 2,2-diphenyl-1-picrylhydrazyl (DPPH) radical scavenging activity in each strains, a modified method from Lin and Chang was employed [44]. In this assay, 0.5 mL of phosphate-buffered saline (PBS) with pH 7.2 was mixed with 0.5 mL of the DPPH solution (0.2 mmol L^−1^ in anhydrous ethanol, Sangon Biotech, Shanghai, China), then incubated in the dark at room temperature for 30 min. A reference control containing distilled water rather than the sample solution was also included. The scavenging activity of DPPH radicals was then monitored by measuring the declining absorbance at 517 nm.

#### 2.3.4. Superoxide Anion Radical (O^2−^) Scavenging Activity

The superoxide anion radical (O^2−^) scavenging activity of the cell-free extract was characterized based on the assay of Liu et al. [47]. First, 0.1 mL of the extract was added to 2.8 mL of Tris-HCl buffer solution (pH 8.2) and 0.1 mL of pyrogallol (0.05 M), then it incubated in water for 20 min at 25 °C. The mixture was then kept in the dark at 25 °C for 4 min. Hydrochloric acid was added to terminate the reaction and the absorbance value was measured at a wavelength of 320 nm. The control, which lacked pyrogallol, was replaced with ultra-pure distilled water.

#### 2.3.5. Hydroxyl Radical (HO•) Scavenging Activity

To measure the hydroxyl radical (HO•) scavenging activity of cell-free extracts, the method of He et al. [48] was used with slight adaptations. The composition of the reaction mixture containing 0.5 mL of the extract, 1 mL of *O*-phenanthroline, 1 mL of PBS (pH 7.2), 0.5 mL of ultra-pure distilled water, 1 mL of 2.5 mmol L^−1^ FeSO_4_, and 1 mL of 20 mmol L^−1^ H_2_O_2_ was incubated for 1.5 h at 37 °C, and the absorbance was then measured at 536 nm.

#### 2.3.6. Reductive Activity

The reductive activity of the Lactobacillus stains was determined using the approach of Lin et al. [49] with slight adaptations according to Tang et al. [50]. The reaction compound consisting of 0.5 mL of cell-free extract, 0.5 mL of potassium ferricyanide, and 0.5 mL of 0.2 M PBS (pH 6.6) was incubated at 50 °C for 20 min. Afterward, 0.5 mL of 10% trichloroacetic acid (TCA) was added, and the mixture was centrifuged at 4500× *g* for 10 min at 4 °C. In this experiment, 1 mL of supernatant was carefully drawn from the uppermost layer. This sample was subsequently mixed with a 0.1% mass fraction solution of ferric chloride (FeCl_3_) and 1 mL of distilled water. The mixture was subjected to spectrophotometric analysis, and its absorbance was measured at a wavelength of 700 nm.

#### 2.3.7. Total Antioxidant Capacity (T-AOC)

Total Antioxidant Capacity (T-AOC) was determined using the Total Antioxidant Capacity (T-AOC) assay kit from Sangon Biotechnology (Shanghai, China) and according to the manufacturer’s instructions. 

### 2.4. In Vitro Probiotics Characteristic Determination

Low pH and Bile Salt Tolerance. In vitro characterization with probiotics involves evaluating the existence and growth of a hypothetical probiotic strain under simulated circumstances that they may encounter in the human gastrointestinal tract. Critical factors to consider in this context: these strains are tolerant to gastric acid and bile salts. The stomach provides a hostile environment with a pH range of 1.0–4.5, and food typically remains in this region for about 3 h [51]. To assess the low pH tolerance of probiotic bacteria, we followed a method similar to the one reported by Argyri et al. [52]. Briefly, the strains were re-suspended in MRS solution (pH was adjusted to 3 with concentrated hydrochloric acid), and samples were withdrawn at 0 and 3 h. Subsequently, samples were diluted and plated onto agar to determine bacterial viability. The small intestine environment, in contrast, is weakly basic with a concentration of bile salts of 0.3% (*w*/*v*). For the assessment of bile salt tolerance of probiotic strains, we followed a modified version of the method described by Sieladie et al. [53]. The putative probiotic strains were injected into MRS solution with 0.3% (*w*/*v*) bile salts, then samples were withdrawn at 0 and 4 h, followed by the enumeration of viable bacteria via plate counting.

Auto-aggregation and cell surface hydrophobicity. To evaluate auto-aggregation, we adopted the method of Del et al. [54] with slight modifications based on Collado et al. [55]. Probiotic strains were grown in MRS solid or liquid medium at 37 °C for 18 h. Afterward, an equal volume of the standardized cell concentration (A_600_ nm = 1 ± 0.05, 10^8^ cells mL^−1^) was harvested with 10 min of centrifugation at 12,000× *g* at 4 °C, washed twice, and re-suspended in their culture supernatant fluid or phosphate-buffered saline (PBS, pH 7.2) for achieving a viable count of about 10^8^ cells mL^−1^. Next, we mixed 4 mL of the cell suspension for 10 s, followed by incubation at room temperature to determine auto-aggregation. We monitored the absorbances of the sample at 0, 2, 4, and 6 h of incubation and measured the absorbed value at 600 nm.

To evaluate cell surface hydrophobicity, we employed a modified version of the methodology as described by Rosenberg et al. [56], with minor adaptations made by other researchers [57,58]. Bacteria were cultured in the liquid medium for 18 h and harvested at rest by centrifugation at 12,000× *g* for 10 min, after which they were washed twice and re-suspended in 0.1 mol mL^−1^ PBS (pH 7.2) at a concentration of roughly 10^8^ CFU mL^−1^. Measurement of the absorbance of the cell suspension was at 600 nm. Subsequently, 1 mL of xylene was added to 3 mL of the cell suspension. After preincubation at room temperature for 10 min, the two-phase system was mixed by vortexing for 2 min. After incubation for 20 min at room temperature, the aqueous phase was then removed to measure its absorbance at 600 nm.

Susceptibility to antibiotics. The susceptibility of the LAB strains was assessed according to the Clinical and Laboratory Standards Institute (CLSI) guidelines for antibiotics [59]. The Kirby Bauer method (disc diffusion method) was applied on Mueller–Hinton agar (MHA) to determine patterns of antibiotic resistance in the selected potential strains. The following antibiotics were tested: 10 mcg of gentamicin, 30 mcg of vancomycin, 15 mcg of erythromycin, 10 mcg of ampicillin, 20 mcg of amoxicillin, 30 mcg of chloramphenicol, 30 mcg of tetracycline, 30 mcg of kanamycin, 30 mcg of clindamycin, and 30 mcg of streptomycin. The antibiotic discs were dispensed onto the solid media and incubated for 48 h at 37 °C under anaerobic conditions. 

Bacterial survival under gastric and intestinal conditions. For the assessment of the survival of these bacteria under gastric and intestinal conditions, simulated gastric and intestinal juices were prepared with hydrochloric acid (pH 1.5–3.0) and bile salt (pH 8.0 ± 0.2) adjusted with 50 mM NaOH, respectively [60]. Bacteria were grown in a liquid medium for 18 h and harvested at the resting period by centrifugation at 12,000× *g* for 10 min, washed twice, and adjusted to roughly 10^8^ CFU mL^−1^. The samples were then diluted into phosphate-buffered saline (0.2 M, pH 7.0). For acid tolerance assays, 1 mL of cell culture was mixed into 9 mL of gastric acid solution (0.32% pepsin and 0.2% NaCl), adapted to pH 3.0, and incubated at 37 °C for 0 and 3 h. The number of cells in the plated layer was then determined. For the simulated intestinal fluid assay, 1 mL of cell culture was mixed into 9 mL of simulated intestinal fluid (0.1% trypsin and 0.3% bile, pH 8.0) and incubated at 37 °C for 0, 3, 6, and 24 h. The plated cell numbers were then determined, and the survival rate was calculated [61]. Altogether, the ability of potential probiotic strains to endure and proliferate under these simulated conditions is a critical criterion for evaluating their potential usefulness as probiotics.

### 2.5. In Vitro Fermentation and Microbial Analysis

Fecal inoculums. The fecal inoculum was prepared using a method based on Zhao et al. [62]. Informed consent forms were signed by four healthy donors, two males and two females, with an average age of 26 ± 1 year, who had not received antibiotic therapy for at least 3 months. The Medical Ethics Committee of the School of Public Health (IRB2201002) approved all procedures. To obtain a 10% (*w*/*v*) fecal suspension, homogenize 100 μL of pooled fecal slurry with sterilized phosphate-buffered saline (pH = 7.4), then filtered through four layers of cheesecloth. The fecal inoculums were collected and processed within an hour.

In vitro intestinal fermentation. For in vitro intestinal fermentation, the basal nutrient medium was prepared following the method by Yu et al. [63] and Fernanda et al. [64]. The pH was adapted to 7.4 with 0.1 M HCl. One milliliter of the fecal slurry was re-suspended in 13 mL of basal medium, and 1 mL of bacteria (10^8^ CFU mL^−1^) was added. The compound was incubated in an anaerobic cabinet at 37 °C for 24 h. Media were subsampled at different time intervals (0, 6, 12, and 24 h) for further analysis, while the medium without bacteria addition served as a negative control. Each fermentation assay was conducted in triplicate.

PCR amplification and high-throughput sequencing. The genomic DNA extracted was obtained from fermentation broth collected at 0 h, 6 h, 12 h, and 24 h using the TIANamp Stool DNA Kit (Tiangen Biotech Co., Ltd., Beijing, China). The DNA yield and purity were assessed using a DNA spectrophotometer (ND-1000, NanoDrop, Wilmington, DE, USA). Extracted genomic DNA was normalized, and a set of primers (F: 5′-CCTACGGGNGGCWGCAG-3′ and R: 5′-GACTACHVGGGTATCTAATCC-3′) was used to perform barcode PCR. The PCR reactions using Phusion High Fidelity PCR Master Mix (Ipswich, Massachusetts, USA) were performed in a volume of 25 μL, using the cycling parameters as follows: 98 °C for 1 min; then, 98 °C for 10 s, 55 °C for 30 s, and 72 °C for 30 s for 30 cycles, and finally extended at 72 °C for 5 min. Following separation with agarose gel electrophoresis and visualization, the PCR products were sequenced on the Illumina NovaSeq platform (Illumina, San Diego, CA, USA).

The samples were identified by paired-ended barcode reading. The paired-end reads were combined with tags by using FLASH (v1.2.11, College Park, Maryland, USA.) after deleting the barcode and primer sequences and the <100 bp reads via TrimGalore. Then, these sequences were aggregated by UPARSE into operational taxonomic units (OTUs) at a 97% similarity cut-off after removing the noise and chimera using the gold.fa database (http://drive5.com/uchime/gold.fa, accessed on 22 February 2023). The rarefaction and alpha were analyzed by Mothur. The bacterial function prediction was carried out using PICRUSt2 (Version 2.1.2-b). The multi-dimensional data from the fermentation broth samples were analyzed by Principal Coordinate Analysis (PCoA) to visualize the differences. The 16S rRNA data are publicly available at the NCBI SRA database (PRJNA936457/936457).

Determination of SCFAs. To determine SCFAs, the fermentation broth was centrifuged and 1 mL of the supernatant solution was mixed with a metaphosphoric acid solution containing an internal standard. After centrifugation and filtration, the supernatant was analyzed by gas chromatography (SP-3420A, Beijing Beifen-Ruili Analytical Instrument Co., Beijing, China) under specific conditions: 90 °C for 1 min, followed by increasing to 120 °C at 10 °C min^−1^ for 1 min, from 120 °C to 150 °C at 10 °C min^−1^, and held at 150 °C for 3 min. The temperature of the spray hole and the auxiliary chamber are both 250 °C [65].

Statistics. Spearman correlation analysis was used to examine the association between various bioactive compounds. One-way ANOVA was used for comparing the SCFAs produced in fermentation, and the nonparametric factorial Kruskal–Wallis rank sum-test was used to check for between-group differences at the bacterial phylum and genus level. Dunn’s test was used for separating means in case significance was found and Tukey-adjusted *p*-values were used for separating means. The authors accepted the statistical significance at *p* < 0.05.

## 3. Result

### 3.1. Higher Antioxidant of P. pentosaceus TCM-3 and L. paracasei TDM-2 Compared with the Reference LAB

**Figure 1:**
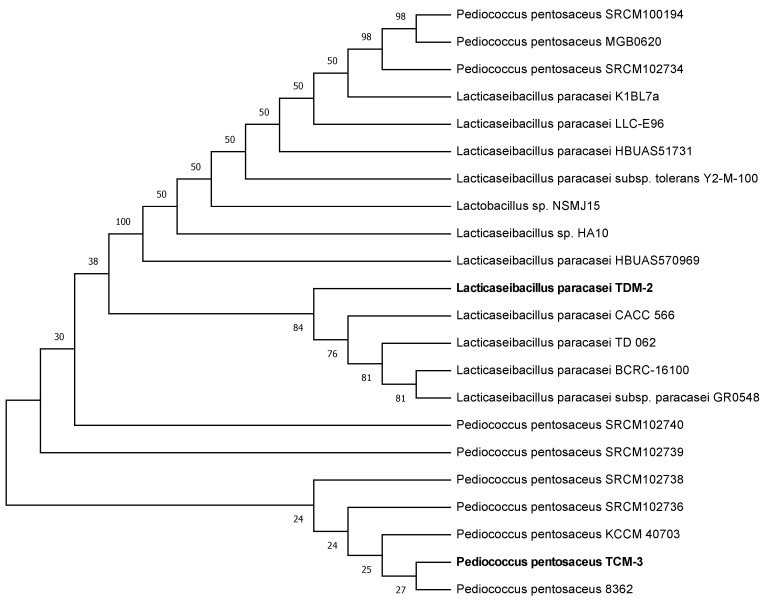
Phylogenetic analysis of 16S rDNA sequences of *Lacticaseibacillus paracasei* TDM-2 and *Pediococcus pentosaceus* TCM-3 with other LAB.

In order to identify potentially probiotic lactic acid bacteria endemic to the Qinghai-Tibetan Plateau, we first isolated strains from 30 dairy products samples using DeMan, Rogosa, and Sharpe Broth, then screened these isolates for antioxidant activity using H_2_O_2_ neutralizing assays. Among 116 isolates, two strains, TCM-3 and TDM-2, showed markedly higher activity than others Table S1. Based on 99–100% nucleotide identity with 16s rRNA gene reference sequences for *Pediococcus pentosaceus* strain 8362 (MT538852) and *Lacticaseibacillus paracasei* strain K1BL7a (MW697451) in the NCBI GenBank database, these strains were designated as *Pediococcus pentosaceus* TCM-3 (OQ449696) and *Lacticaseibacillus paracasei* TDM-2 (OQ449697) (Figure 1).

We then conducted a battery of in vitro antioxidant activity assays to compare the activities of TDM-2 and TCM-3 with that of the well-established LAB reference probiotic strain, *Lactobacillus rhamnosus* CICC 6001 (LGG), including DPPH radical scavenging activity, resistance to H_2_O_2_, superoxide anion radical (O^2−^) scavenging activity, reductive activity, hydroxyl radical (HO•) scavenging activity, and total antioxidant capacity (T-AOC) (Table 1). In DPPH radical scavenging assays, TCM-3 showed 87.99% free radical scavenging activity compared to reactions without cells, whereas TDM-2 displayed 84.15% inhibition and LGG showed 84.13% inhibition. We then compared the viability of these strains following exposure to H_2_O_2_ at a range of concentrations. After incubation in 2.0 mM H_2_O_2_, 63.65% of TDM-2 remained viable, while TCM-3 showed 71.79% resistance to H_2_O_2_, and LGG was 53.17% resistant. The results of HO• radical scavenging activity indicated that the LAB strains showed diverse scavenging activities in the sequence as follows: TDM-2 (92.33 ± 6.19), TCM-3 (97.00 ± 5.67), and LGG (84.02 ± 5.36). In O^2−^ radical scavenging assays, TCM-3 showed significantly higher scavenging activity than either TDM-2 or LGG (37.59% vs. 17.17% and 26.92%, respectively; *p* < 0.05). While reduction assays indicated that TDM-2 had the highest reductive activity (29.48%, *p* < 0.05), followed by TCM-3, then LGG (13.38% and 10.20%, respectively, *p* < 0.05). Finally, total antioxidant capacity (T-AOC) measurements showed that TDM-2 had higher T-AOC activity than either TCM-3 or LGG (4.10 μmol mL^−1^ vs 3.68 and 3.53 μmol mL^−1^, respectively, *p* < 0.05). These findings suggest that strains TDM-2 and TCM-3 had comparable antioxidants as free radical scavenging activity to that of strain LGG by standard in vitro assays.

### 3.2. Resistance to Acid and Bile Salt under Gastric and Intestinal Conditions

The selected cultures were further examined for observing their survival rates under artificial gastric acid (pH 3.0, pepsin for 3 h) and bile salt (0.3% *w*/*v* bovine salt with trypsin for 4 h) in order to identify whether potential probiotic bacteria can tolerate acids and pass through the intestinal tract (Table 2). Under acidic conditions (pH 3), the LAB strains showed obvious differences (*p* < 0.05). The survival rate of TDM-2 and LGG reached 104.88% and 104.48%, which is higher than that of TCM-3 as 97.26% (*p* < 0.05). 

At the highest bile salt concentration (Table 2). To be specific, the survival rate of TDM-2 was 121.19%, while slightly lower survival rates of LGG and TCM-3 as 108.22% and 107.46% were presented (*p* < 0.05), respectively, and collectively suggested that the selected LAB strains were well tolerated to acid and bile salts. 

In vitro gastric and intestinal fluid tolerance culture methods were further applied to determine the ability of LAB strains to tolerate gastric and intestinal conditions and thus survive as live probiotics in the intestinal tract (Table 2). Inspection of strain TCM-3 and TDM-2 viability following gastric juice exposure demonstrated that the TDM-2 showed a slightly higher survival rate (113.31%), following with TCM-3 and LGG (97.98% and 101.28%) after incubation at pH 3.0 for 3 h. In addition, incubation with intestinal juice media at 3, 6, and 24 h showed that the number of cells did not decrease after 3 h incubation. Specifically, the survival rates of TDM-2 and TCM-3 were 118.57% and 108.38% respectively, and decreased slightly after 6 h (108.33% and 103.27%), and after 24 h, a range of 94.79–96.20% for selected LAB strains survived intestinal juice treatment.

### 3.3. Auto-Aggregation and Cell Surface Hydrophobicity

Auto-aggregation and hydrophobicity were analyzed in order to identify the probiotic’s roles in preventing pathogens from forming biofilms and adhering to intestinal mucus (Table 2). Strain TDM-2 exhibited an auto-aggregation potential of 86.26–93.55% within a 2 h to 6 h incubation period, following with the strain TCM-3 as 72.26% to 87.12%, while the lowest auto-aggregation potential was presented by strain LGG as 70.67% to 86.01% (*p* < 0.05). Additionally, the cell surface hydrophobicity of LAB strains was highly variable (26.03–86.84%) and thus suggested different bacterial adhesion potential, in which, TDM-2 exhibited the highest cell surface hydrophobicity, while LGG exhibited the lowest value (86.84% vs. 26.03%, *p* < 0.05).

### 3.4. Susceptibility to Antibiotics

The selected strains TDM-2 and TCM-3 were inspected for antibiotic susceptibility to confirm their safety (Table 3). The results indicated that strain TCM-3 was susceptibility to vancomycin, ampicillin, gentamicin, clindamycin, erythromycin, tetracycline, amoxicillin, chloramphenicol, and streptomycin. TDM-2 was susceptibility to vancomycin, ampicillin, gentamicin, clindamycin, erythromycin, tetracycline, amoxicillin, chloramphenicol, and kanamycin. Therefore, ingestion of strain TDM-2 and TCM-3 could be safe against antibiotic susceptibility problems.

### 3.5. Addition of TCM-3 or TDM-2 Significantly Alters Microbiota Structure In Vitro

To further research the influence of supplementing TCM-3 or TDM-2 on gut microbial composition and diversity, we conducted metagenomic analysis of human gastrointestinal tract microbiota (obtained from feces of healthy volunteers) cultured in bioreactors in vitro, with or without added TCM-3 or TDM-2. Comparison of Alpha diversity, i.e., taxonomic richness and diversity, through Observed, Chao1, and Shannon indices at 24 h of fermentation (Figure S1A) showed that microbiota supplemented with TDM-2 had lower species richness than fermentations with TCM-3 or neither strain (controls). In contrast, there were no significant differences observed in the Shannon index between the groups.

**Figure 2:**
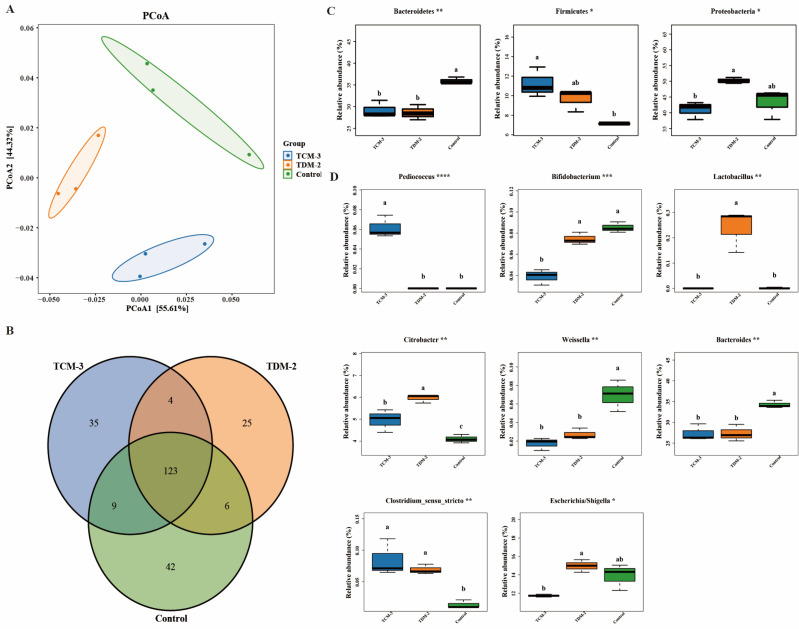
Comparison of bacterial beta diversity, unique taxa, and community composition among groups after 24 h of fermentation in vitro. (**A**) Weighted UniFrac principal coordinate analysis (PCoA) of human gut microbiota in fermentations supplemented or not with TDM-2, TCM-3. (**B**) Venn diagram of unique and shared OTUs between TCM-3, TDM-2, and control groups. (**C**,**D**) Boxplots of differential phyla (**C**) and species (**D**) between fermentation groups; significant differences in the abundance of each taxa were determined by the nonparametric factorial Kruskal–Wallis rank level of * (*p* < 0.05), ** (*p* < 0.01), *** (*p* < 0.001), **** (*p* < 0.0001) with lowercase letters indicating differences between groups.

We then used principal coordinate analysis (PCoA) on the basis of a weighted UniFrac distance matrix to examine whether and how the addition to TCM-3 or TDM-2 affected beta diversity of gut microbiota in vitro. At 24 h of fermentation, communities in the TCM-3, TDM-2, and control groups showed obvious separation (Figure 2A), with the PCoA1 axis explaining 55.61% of the observed variation and the PCoA2 axis explaining approximately 44.32% of the observed variation among samples. We also noted that replicate samples from microbiota supplemented with either TCM-3 or TDM-2 showed obviously tighter clustering than those from the control fermentation containing neither strain. PERMANOV (Figure S1B) analysis further supported the significance of these differences in beta diversity among groups (*p* < 0.001).

Sequence analysis identified a total of 244 OTUs, 123 of which were detected in all three groups at 24 h of fermentation (Figure 2B). By contrast, 42, 35, and 25 unique OTUs were found in the control, TCM-3, and TDM-2 samples, respectively. LEfSe analysis of potential indicator taxa under different treatments identified 10 taxa significantly associated with control communities (including Clostridiales and Bifidobacteriales on the Order level, *Bifidobacteriaceae* and *Leuconostocaceae* at the Family level, and *Bacteroides caccae*, *Bacteroides_stercoris*, *Bacteroides_caccae*, and *Bifidobacterium_adolescentis* on the species level), 5 taxa associated with TDM-2 supplementation (including Order *Lactobacillales*, *Class Bacilli*, Family *Lactobacillaceae*, etc.), and 2 taxa associated with TCM-3 samples (i.e., the *Pediococcus* and *Blautia* genera) (Figure S1C). These results suggested that the addition of these two strains resulted in enrichment for different taxa which altered the overall community diversity.

In light of these effects on diversity and indicator taxa by TCM-3 or TDM-2, we next explored specific differences in bacterial community composition between fermentations of these human microbiota. At the Phylum level, Proteobacteria (41.02–50.22%) and Bacteroidetes (28.62–35.94%) were the dominant clades in all three groups (Figure S2A), with Fusobacteria (11.28–18.50%) and Firmicutes (7.12–11.23%), including the next most abundant taxa after 24 h of fermentation. These four phyla with the highest relative abundance together of all bacterial OTUs accounted for over 90%. At 24 h of fermentation, significant differences in community composition among groups generally corresponded to change in the abundance of *Bacteroidetes* and *Firmicutes* (Figure 2C), with *Bacteroidetes* significantly decreasing in the presence of TCM-3 or TDM-2 (*p* < 0.05). By contrast, *Firmicutes* increased in abundance following addition of either LAB strain (*p* < 0.05). Furthermore, Proteobacteria were enriched in the presence of TDM-2 (*p* < 0.05) in comparison to their levels in the TCM-3 and control fermentations.

On the genus level, the dominating genera were *Bacteroides* (27.32–34.28%) and *Fusobacterium* (11.28–11.50%) in all samples (Figure S2B). However, *Pediococcus* had higher abundance and *Bifidobacterium* had lower abundance in fermentations supplemented with TCM-3 compared to TDM-2 and control samples (*p* < 0.05) (Figure 2D). In fermentations treated with TDM-2, *Lactobacillus* and *Citrobacter* were more abundant than in cultures supplemented with TCM-3 or control samples (*p* < 0.05). In control samples without TCM-3 or TDM-2, *Weissella* and *Bacteroides* were noticeably higher (*p* < 0.05), whereas *Clostridium*_*sensu_stricto* were both decreased in the control group compared to ferments containing TDM-2 or TCM-3 (*p* < 0.05) groups. In addition, we also noted that *Escherichia*/*Shigella* was less abundant in fermentations treated with TCM-3 than in those with added TDM-2 (*p* < 0.05).

### 3.6. Pediococcus Is Positively Correlated with SCFA Contents

Based on the above metagenomic evidence of significant structural shifts in microbiota associated with TCM-3 or TDM-2, we next investigated possible functional effects of these strains. To this end, we used GC-MS to quantify short-chain fatty acid (SCFA) production in fermentations with or without each of these LAB strains (Table 4) (Figures S3–S5). In general, fermentations of human microbiota supplemented with TCM-3 had obviously higher contents of acetic acid, propionic acid, and total SCFAs compared to those with TDM-2 or the control group at 12 h and 24 h (*p* < 0.05) and propionic acid enrichment in the TCM-3 group beginning at 12 h. These results supported the inference that the addition of TCM-3 in human gut microbiota could lead to increased SCFA production.

**Figure 3:**
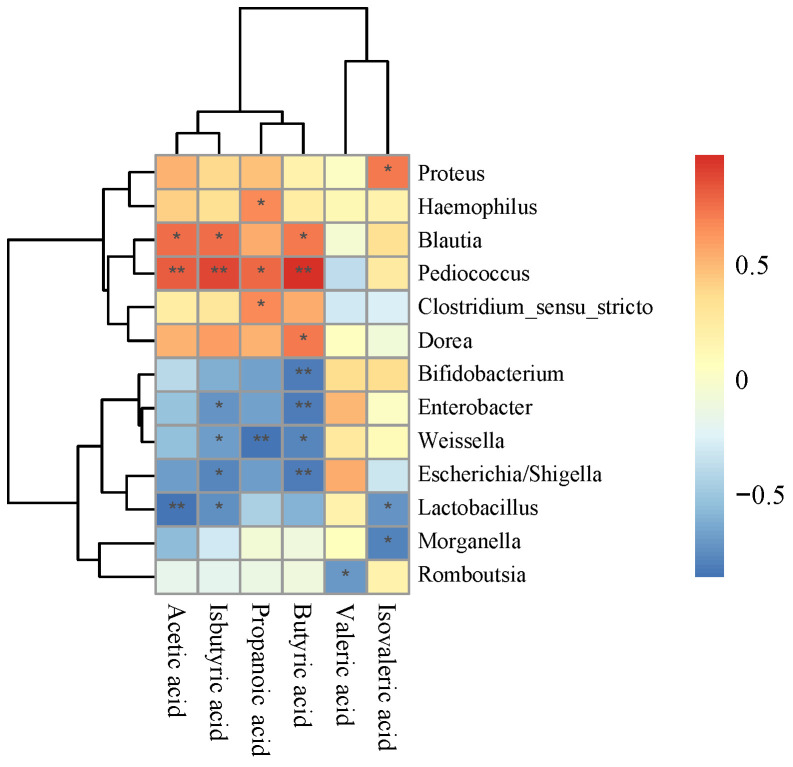
Heatmap of SCFA contents correlated with various bacterial taxa. Correlation Heatmap analysis calculates the correlation coefficient (R value) and significance level (*p* value) between each SCFA and species. The color depth indicates the size of the R value, the legend is the color interval for different R values. Positive correlations are represented by red color and negative correlations by blue color. Color intensities are proportional to the correlation coefficients (Spearman correlation). Significant correlations are indicated by * (*p* < 0.05), ** (*p* < 0.01).

Based on the above differences in microbial taxa associated with TCM-3 or TDM-2 addition, combined with differences in SCFA production among fermentation groups, we then performed Spearman correlation analysis in order to identify whether and which taxa might be correlated with specific SCFAs (Figure 3). This analysis indicated that *P. pentosaceus* TCM-3 was positively associated with acetate, propionate, butyrate, and isobutyrate densities (r > 0.5, *p* < 0.05); *Blautia* showed a positive correlation with acetate, butyrate, and isobutyrate concentrations (r > 0.5, *p* < 0.05); and *Clostridium_sensu_stricto* was positively correlated with propionate densities (r > 0.5, *p* < 0.05). Furthermore, *Weissella*, *Bifidobacterium*, *Enterobacter*, *Escherichia*/*Shigella*, and *Lactobacillus* were significantly negatively associated with butyrate content (r < −0.5, *p* < 0.05). These results aligned well with our above community composition analysis showing enrichment for genus *Blautia* and *Clostridium_sensu_stricto* in TCM-3 fermentations, along with decreased levels of Proteobacteria, such as *Escherichia*/*Shigella* and *Weissella*. Taken together, these findings implied that the supplementation with TCM-3 could lead to enhanced SCFA production along with enrichment for specific taxa in human gut microbiota.

## 4. Discussion

In recent years, strategies employing probiotics to alter intestinal microbiota and ameliorate inflammation induced by oxidative stress have received increasing research attention. The Qinghai-Tibetan plateau is well-established as an extreme environment due its high altitude, low temperature, and high UV conditions, leading to oxidative stress. Thus, potentially commensal microbes, such as LAB, isolated from this environment might serve as effective probiotics for ameliorating oxidative stress [26]. In the current study, we identified *P*. *pentosaceus* TCM-3 and TDM-2 as potential LAB strains from the Qinghai-Tibetan Plateau based on their relatively high antioxidant effects in vitro. Physiological characterization regarding their tolerance to low pH and high bile conditions supported the likelihood that these strains settle in the gastrointestinal tract of mammals. Testing their potential probiotic characteristics, we also added these respective strains to in vitro fermentations of human gut microbiota and found that these strains not only enrich for specific taxa in metagenomic analysis, but also that SCFA production, a hallmark of probiotic activity, is significantly higher in the presence of TCM-3.

In inflammatory diseases of the gastrointestinal tract, ROS accumulation accompanies impaired Redox homeostasis and cellular damage. We therefore examined whether LAB strains TCM-3 and TDM-2 could directly exhibit antioxidant activity to counteract or neutralize ROS in vitro through DPPH radical scavenging, H_2_O_2_ resistance, reduction, HO• scavenging activity, O^2−^ scavenging activity, and T-AOC estimation, using the well-known LAB strain LGG as a control. Although the antioxidant activity of these strains was generally high, comparable to that of LGG, their effects obviously differed between assays, suggesting that each strain might employ different cellular mechanisms to alleviate ROS. Moreover, the intestinal epithelial cells produce and release large amounts of ROS under excessive pathogen proliferation in the intestine, and microbes in the gut have been shown to modulate host redox signaling and homeostasis [66]. Thus, probiotic treatment with LAB strains might impact oxidative stress in the host both directly, through ROS scavenging, and indirectly, by altering the community structure of intestinal microbiota [67]. It is therefore possible that the direct antioxidant activity by TCM-3 and TDM-2 might confer probiotic effects by modulating host response or by modulating conditions in the gut microenvironment to promote enrichment of beneficial rather than pathogenic functional microbiota.

Probiotic strains are necessarily tolerant of acidic conditions and stomach bile to remain viable and colonize the lower gastrointestinal tract after ingestion [68]. Bacteria exposed to low pH typically show markedly reduced viability [69,70], and survival rates under acidic conditions can vary widely among probiotic strains [71]. In this study, TCM-3 and TDM-2 showed relatively high tolerance to acid and bile, suggesting that they could remain viable in the gastrointestinal [72,73]. In addition, TDM-2 showed intense auto-aggregation and had high cell surface hydrophobicity, further supporting a potential probiotic role in preventing adhesion to intestinal mucus and biofilm formation by pathogens. Furthermore, TDM-2 and TCM-3 showed susceptibility to antibiotics, implying that these strains are unlikely to serve as a source of antibiotic resistance, further highlighting their probiotic potential.

Recent clinical evidence has emerged showing that probiotics function in modifying the gut microbiome among gastrointestinal diseases, such as inflammatory bowel disease. Miele and colleagues reported that a concentrated blend of probiotic bacterial strains with high antioxidative activity could effectively maintain relief with ulcerative colitis in children [74]. Based on their strong antioxidant capacity in vitro, we thus speculated that the TCM-3 and TDM-2 strains could potentially modulate gut microbiota. Although we found no significant effects on Shannon index after 24 h of fermentation with human gut microbiota in vitro, PCoA analysis revealed significant separation of fermentation communities supplemented with TCM-3, TDM-2, or neither, suggesting that these strains can indeed shift the overall composition of gut microbiota.

Phylum level analysis showed that adding either one of these strains leads to significant enrichment with Firmicutes but lower levels of Bacteroidetes, both of which are predominant phyla in the healthy human gut. Firmicutes in the gut reportedly harbor genes, such as AMPK or GLP1, that mediate fermentation of dietary fiber, and that Firmicutes may interact with intestinal mucosa, potentially contributing to maintenance of intestinal homeostasis [75]. The Firmicutes/Bacteroidetes ratio has garnered considerable attention as an increasingly accepted indicator of intestinal health or dysbiosis. Specifically, a low ratio is associated with dysbiosis in inflammatory bowel disease, whereas probiotic strategies target this balance between Firmicutes and Bacteroidetes to confer health benefits to the host [76]. Notably, fermentations treated with *L. paracasei* TDM-2 show marked enrichment for Proteobacteria, which could reduce oxygen levels and lower redox potential in the gut, facilitating colonization by strict anaerobes required for healthy gut function [77].

At the genus level, fermentations with *P. pentosaceus* TCM-3 have increased *Pediococcus* and reduced *Bifidobacterium* abundance, while *Lactobacillus* is more abundant in fermentations with *L. paracasei* TDM-2, which could drive enrichment for other LAB strains in the gut. Huang et al. demonstrated that *Pediococcus* can improve gastrointestinal motility and promote the production of beneficial bacteria that generate short-chain fatty acids [78]. Zhou et al. also reported that *Lactobacillus* can attenuate cytotoxicity and intestinal barrier damage induced by *Clostridium perfringens* [79]. Furthermore, the abundance of *Clostridium_sensu_stricto* was higher in fermentations treated with TCM-3 or TDM-2 than in the control group, which has also been reported to enhance short-chain fatty acid production [80].

Gut microbiota can also influence host health by secreting various metabolites. In particular, SCFAs, for example, acetic and propanoic acids and butyric acids, are among the main products of bacterial fibrous fermentation in the gut. SCFAs reportedly stimulate maintenance of intestinal barrier completeness, mucus generation, and defense against inflammation to improve gut health [81]. In the present study, we analyzed changes in SCFA production associated with the addition of TCM-3 or TDM-2 to investigate their possible metabolic effects. Acetic acid, propionic acid, and total SCFAs were higher for fermentations with TCM-3 compared to those with TDM-2 or control fermentations, supporting that TCM-3 could potentially enhance SCFA production in human gut microbiota. The production of SCFAs, such as acetic, propionic, and butyric acids, has been linked to the abundance of specific taxa in the intestinal tract, such as commensal *Clostridium* spp. and *Ruminococcus* spp. [80,82], aligning well with the positive correlation between *Clostridium_sensu_stricto* propionate in the current work. In a previous study, *P. pentosaceus* CECT 8330 was correlated with higher fecal SCFA levels [83], which was also in agreement with our correlation analysis linking *Pediococcus* abundance with acetate, propionate, butyrate, and isobutyrate concentrations.

## 5. Conclusions

Overall, the *P. pentosaceus* TCM-3 and *L. paracasei* TDM-2 strains isolated from dairy produced on the Qinghai-Tibetan plateau display high antioxidant capacity and probiotic properties associated with enrichment for specific taxa and elevated SCFA production. These findings support the further exploration of these strains for potential development as therapeutic probiotic interventions to promote gut health. Further, the regular inclusion of probiotics in our daily diet may provide a practical and cost-efficient strategy for replenishing any potential diminutions in our gut microbiota and improve our health.

## Figures and Tables

**Table 1:** Antioxidant activity of strain TDM-2 and TCM-3.

	TDM-2	TCM-3	LGG
DPPH free radical scavenging activity (%)	84.15 ± 0.03 ^b^	87.99 ± 0.07 ^a^	84.13 ± 0.02 ^b^
H_2_O_2_-resistant ability (%)			
0 mmol L^−1^ H_2_O_2_	77.70 ± 0.09 ^b^	89.19 ± 0.08 ^a^	67.40 ± 0.02 ^c^
1.0 mmol L^−1^ H_2_O_2_	73.43 ± 0.03 ^b^	84.92 ± 0.01 ^a^	55.40 ± 0.10 ^c^
1.5 mmol L^−1^ H_2_O_2_	72.74 ± 0.06 ^b^	79.70 ± 0.07 ^a^	53.96 ± 0.06 ^c^
2.0 mmol L^−1^ H_2_O_2_	63.65 ± 0.04 ^b^	71.79 ± 0.09 ^a^	53.17 ± 0.06 ^c^
HO• scavenging activity (%)	92.33 ± 0.09 ^b^	97.00 ± 0.07 ^a^	84.02 ± 0.06 ^b^
O^2−^ scavenging activity (%)	17.17 ± 0.08 ^c^	37.59 ± 0.02 ^a^	26.92 ± 0.02 ^b^
Reductive activity (%)	29.48 ± 0.03 ^a^	13.38 ± 0.00 ^b^	10.20 ± 0.01 ^c^
T-AOC (μmoL/mL)	4.10 ± 0.17 ^a^	3.68 ± 0.08 ^b^	3.53 ± 0.02 ^b^

The values are means ± SD (n = 3) of from triplicate of independent experiments, and the alphabetic superscripts (a, b, c) represented in the same columns followed by different letters were significantly different, based on the one-way ANOVA test (*p* < 0.05).

**Table 2:** Probiotic attributes for selected lactic acid bacteria.

		TDM-2	TCM-3	LGG
Survival Rate of Acid after 3 h (%)	pH 3	104.88 ± 0.76 ^a^	97.26 ± 0.49 ^b^	104.48 ± 0.81 ^a^
Survival Rate of Bile after 4 h (%)	pH 8	121.19 ± 0.69 ^a^	107.46 ± 0.52 ^b^	108.22 ± 0.04 ^b^
Gastric juice	3 h	113.31 ± 0.12 ^a^	97.98 ± 0.25 ^b^	101.28 ± 1.10 ^b^
Intestinal juice	3 h	118.57 ± 0.78 ^a^	108.38 ± 0.93 ^b^	103.93 ± 0.44 ^c^
	6 h	108.33 ± 1.21 ^a^	103.27 ± 0.27 ^a^	94.48 ± 0.77 ^b^
	24 h	94.79 ± 0.10 ^b^	96.20 ± 0.18 ^a^	87.05 ± 0.17 ^b^
Auto-aggregation (%)	0 h	64.72 ± 0.28 ^b^	68.75 ± 0.08 ^c^	66.98 ± 0.13 ^a^
	2 h	86.26 ± 0.14 ^a^	72.26 ± 0.01 ^c^	70.67 ± 1.43 ^b^
	4 h	93.08 ± 0.07 ^a^	77.72 ± 0.01 ^c^	81.88 ± 0.03 ^b^
	6 h	93.55 ± 0.01 ^a^	87.12 ± 0.04 ^c^	86.01 ± 0.01 ^b^
Hydrophobicity (%)		86.84 ± 0.21 ^a^	30.82 ± 0.07 ^b^	26.03 ± 0.10 ^b^

The values represented are the mean ± SD (n = 3) of survival percentage of the bacterial cultures used, and the alphabetic superscripts (a, b, c) represented in the same columns followed by different letters were significantly different, *p* values are based on the one-way ANOVA test (*p* < 0.05).

**Table 3:** Antibiotic sensitivity of strain TDM-2 and TCM-3.

	TDM-2	TCM-3
Antibiotic Susceptibility		
VAN	S	S
AM	S	S
CN	S	S
CLI	S	S
ERY	S	S
TE	S	S
AMC	S	S
CPL	S	S
KAN	S	R
SM	R	S

The resistance (R), and susceptibility (S); VAN: vancomycin; AM: ampicillin; CN: gentamicin; CLI: clindamycin; ERY: erythromycin; TE: tetracycline; AMC: amoxicillin; CPL: chloramphenicol; KAN: kanamycin; SM: streptomycin.

**Table 4:** Fatty acid contents in fermentations with or without TDM-2 or TCM-3.

SCFAs	Time of Incubation (h)	TCM-3 (mmol/L)	TDM-2 (mmol/L)	Control (mmol/L)
Acetic acid	0 h	3.18 ± 0.22 ^a^	2.71 ± 0.04 ^b^	2.83 ± 0.05 ^b^
	6 h	3.82 ± 0.01 ^a^	3.61 ± 0.01 ^b^	3.64 ± 0.04 ^b^
	12 h	4.54 ± 0.09 ^a^	4.06 ± 0.03 ^b^	4.00 ± 0.05 ^b^
	24 h	4.99 ± 0.02 ^a^	4.90 ± 0.08 ^b^	4.82 ± 0.09 ^b^
Propanoic acid	0 h	0.88 ± 0.00 ^a^	0.88 ± 0.01 ^a^	0.88 ± 0.01 ^a^
	6 h	0.97 ± 0.00 ^a^	0.956 ± 0.00 ^b^	0.96 ± 0.01 ^b^
	12 h	1.24 ± 0.02 ^a^	1.06 ± 0.03 ^b^	1.05 ± 0.02 ^b^
	24 h	1.35 ± 0.01 ^a^	1.24 ± 0.02 ^b^	1.22 ± 0.01 ^b^
Butyric acid	0 h	0.50 ± 0.00 ^a^	0.05 ± 0.00 ^a^	0.049 ± 0.00 ^a^
	6 h	0.49 ± 0.00 ^a^	0.05 ± 0.00 ^a^	0.049 ± 0.00 ^a^
	12 h	0.49 ± 0.00 ^a^	0.05 ± 0.00 ^b^	0.049 ± 0.00 ^b^
	24 h	0.05 ± 0.00 ^a^	0.05 ± 0.00 ^b^	0.049 ± 0.00 ^b^
Valeric acid	0 h	0.10 ± 0.00 ^a^	0.10 ± 0.00 ^a^	0.10 ± 0.00 ^a^
	6 h	0.11 ± 0.01 ^a^	0.12 ± 0.01 ^a^	0.12 ± 0.01 ^a^
	12 h	0.11 ± 0.01 ^a^	0.11 ± 0.01 ^a^	0.11 ± 0.01 ^a^
	24 h	0.10 ± 0.00 ^a^	0.11 ± 0.01 ^a^	0.11 ± 0.01 ^a^
Isobutyric acid	0 h	0.40 ± 0.00 ^a^	0.40 ± 0.00 ^a^	0.40 ± 0.00 ^a^
	6 h	0.40 ± 0.00 ^a^	0.40 ± 0.00 ^a^	0.40 ± 0.00 ^a^
	12 h	0.40 ± 0.00 ^a^	0.40 ± 0.00 ^b^	0.40 ± 0.00 ^ab^
	24 h	0.42 ± 0.00 ^a^	0.41 ± 0.01 ^a^	0.42 ± 0.01 ^a^
Isovaleric acid	0 h	0.059 ± 0.00 ^a^	0.058 ± 0.00 ^a^	0.06 ± 0.00 ^a^
	6 h	0.057 ± 0.00 ^a^	0.058 ± 0.00 ^a^	0.06 ± 0.00 ^a^
	12 h	0.058 ± 0.00 ^a^	0.058 ± 0.00 ^b^	0.06 ± 0.00 ^ab^
	24 h	0.06 ± 0.00 ^a^	0.058 ± 0.00 ^b^	0.06 ± 0.00 ^b^
Total fatty acids	0 h	4.67 ± 0.22 ^a^	4.21 ± 0.04 ^b^	4.34 ± 0.07 ^b^
	6 h	5.41 ± 0.02 ^a^	5.20 ± 0.01 ^b^	5.22 ± 0.04 ^b^
	12 h	6.40 ± 0.10 ^a^	5.74 ± 0.01 ^b^	5.68 ± 0.07 ^b^
	24 h	6,97 ± 0.02 ^a^	6.67 ± 0.10 ^b^	6.68 ± 0.11 ^b^

Data are expressed as means ± standard deviations (n = 3). Different superscript lowercase letters (a, b within the same row indicate differences in significance at *p* < 0.05. *p* values are based on the one-way ANOVA test.

## Data Availability

The data presented in this study are available on request from the corresponding author.

## Supplementary Materials

The following supporting information can be downloaded at: https://www.mdpi.com/article/10.3390/foods12091814/s1, Figure S1: Analysis of Alpha diversity for bacterial communities; Figure S2: Effects of TDM-2 and TCM-3 supplementation on gut microbiota diversity; Figure S3: The GC-MS separation of fatty acid of *Lacticaseibacillus paracasei* TDM-2; Figure S4: The GC-MS separation of fatty acid of *Pediococcus pentosaceus* TCM-3; Figure S5: The GC-MS separation of fatty acid of control; Table S1: H_2_O_2_-resistant ability of all strains (%).
