# Supplementary material for: High Antioxidant Capacity of Lacticaseibacillus paracasei TDM-2 and Pediococcus pentosaceus TCM-3 from Qinghai Tibetan Plateau and Their Function towards Gut Modulation
Source: Foods. 2023 Apr 27;12(9):1814. doi: 10.3390/foods12091814 (PMC10178559; doi:10.3390/foods12091814)
Supplement: Supplementary file 1 [file foods-12-01814-s001.zip › foods-2324950-supplementary.pdf]

Supplementary table

Supplementary Table S1. H_2O_2-resistant ability of all strains (%)

	0 mmol/L	1 mmol/L	1.5 mmol/L	2 mmol/L
4	57.27±2.00	56.23±0.37	54.95±3.32	53.35±1.27
5	35.93±2.08	30.01±0.70	20.68±0.21	18.74±0.12
6	68.43±0.69	67.79±1.12	67.46±1.13	65.37±0.68
8	83.81±0.59	67.02±2.57	67.02±2.57	60.48±0.06
11	20.67±0.27	19.66±0.51	19.02±0.02	17.99±0.01
12	50.75±0.08	46.74±1.31	24.62±0.09	22.61±0.50
13	89.58±0.64	86.45±0.13	65.92±0.61	64.92±1.27
15	47.22±0.39	47.01±0.17	24.94±1.00	24.32±0.07
16	23.25±0.02	22.37±0.08	22.06±0.26	16.84±0.78
17	49.53±0.05	48.25±0.20	46.77±1.28	45.47±0.36
18	48.15±0.30	46.87±0.66	24.44±0.79	23.79±0.08
20	48.55±0.01	44.42±0.35	24.40±1.14	23.79±0.08
21	64.97±0.06	59.37±0.02	58.94±0.16	57.49±0.16
22	22.01±0.02	20.39±0.09	17.67±0.04	19.86±0.02
23	55.76±0.34	52.55±0.02	51.79±0.45	48.20±0.98
24	68.11±0.88	66.56±0.05	47.17±0.83	45.53±0.50
30	69.21±0.14	68.96±0.44	66.98±0.16	65.91±0.03
31	21.68±0.02	20.61±0.02	19.65±0.03	17.62±0.04
32	54.65±1.11	54.75±0.09	52.47±0.35	51.68±0.84
34	54.81±0.63	52.68±0.73	51.87±0.11	51.81±0.36
35	60.57±0.13	59.09±0.99	56.27±0.27	54.81±0.58
36	69.21±0.01	68.33±0.07	68.02±0.08	65.11±0.06
37	57.15±0.02	57.34±0.01	57.64±0.05	55.31±0.05
41	71.33±0.06	70.54±0.02	68.94±0.02	68.28±0.01
42	23.82±0.08	21.14±0.05	19.53±0.09	17.44±0.02
43	61.40±0.04	56.30±0.03	56.19±0.06	55.56±0.03
44	25.49±0.09	25.40±0.04	23.38±0.02	18.50±0.09
45	55.33±0.02	53.11±0.06	48.87±0.04	43.02±0.01
46	69.42±0.03	68.42±0.02	67.95±0.02	67.87±0.03
47	66.54±0.04	65.23±0.03	62.54±0.02	60.57±0.03
48	69.90±0.01	68.04±0.02	67.56±0.07	65.82±0.02
49	24.12±0.01	19.34±0.06	18.02±0.02	17.13±0.03
50	70.23±0.01	67.68±0.05	67.27±0.02	66.45±0.06
51	74.53±0.88	70.59±0.95	51.32±0.00	44.28±0.65
52	68.89±0.05	68.36±0.02	67.78±0.03	65.29±0.05
53	71.16±0.36	69.53±0.14	67.86±0.13	50.84±0.28
54	64.89±0.37	62.65±0.08	62.46±0.06	59.20±0.05
55	68.86±0.37	65.20±0.29	61.92±0.24	60.94±0.65
57	55.90±0.62	50.65±0.66	45.76±0.88	44.78±0.62
58	22.07±0.10	21.61±0.08	21.60±0.01	17.38±0.07

61	66.72±0.08	63.70±0.23	43.07±0.02	38.71±0.04
65	81.22±0.40	79.78±0.06	64.26±0.06	61.91±0.02
66	69.67±0.57	68.76±0.63	50.02±0.58	48.01±0.33
68	70.89±0.29	58.55±0.05	58.92±0.04	57.89±0.07
70	35.01±0.01	32.61±0.22	29.13±0.02	20.42±0.03
71	47.06±0.34	45.23±0.08	23.92±0.16	23.18±0.12
72	48.35±0.02	46.84±0.06	26.23±0.01	24.11±0.01
73	37.46±0.13	30.15±0.05	27.85±0.07	24.92±0.27
74	28.42±0.02	26.57±0.09	23.07±0.16	18.93±0.08
75	72.07±0.18	66.31±0.31	55.28±0.64	50.82±0.48
76	41.08±0.06	40.09±0.02	40.06±0.01	34.49±0.07
78	24.07±0.06	22.23±0.07	20.62±0.05	19.54±0.05
80	51.92±0.16	49.30±0.15	28.58±0.11	24.53±0.06
81	46.85±0.11	45.95±0.23	44.82±0.01	42.73±0.12
83	69.06±0.14	68.56±0.21	67.66±0.05	64.26±0.13
84	68.45±0.40	68.16±0.04	68.07±0.07	65.18±0.20
85	49.95±0.09	49.33±0.01	47.93±0.17	47.46±0.07
90	52.83±0.70	51.94±0.24	49.95±0.28	46.54±0.33
92	49.16±0.02	47.18±0.07	26.46±0.03	23.41±0.03
94	57.16±0.03	51.76±0.03	28.43±0.12	24.66±0.01
95	24.58±0.03	22.10±0.06	20.86±0.07	17.48±0.27
96	23.39±0.20	21.82±0.13	20.21±0.08	17.76±0.10
97	47.86±0.14	43.91±0.05	26.79±0.02	24.02±0.09
98	66.06±0.21	65.49±0.12	64.95±0.31	64.95±0.31
99	65.25±0.07	63.20±0.05	62.69±0.01	61.89±0.08
101	84.22±0.99	81.37±0.38	65.89±0.64	61.78±0.13
103	30.95±0.14	27.25±0.12	22.55±0.03	19.61±0.07
104	42.14±0.11	40.80±0.32	40.72±0.17	38.25±0.11
105	32.03±0.58	31.49±0.14	22.72±0.27	22.27±0.01
106	49.15±0.05	47.03±0.03	27.21±0.02	24.03±0.01
107	51.19±0.14	48.64±0.02	27.35±0.01	23.08±0.03
108	71.29±0.02	70.59±0.15	70.16±0.31	68.09±0.02
111	69.09±0.15	68.31±0.05	64.74±0.18	64.19±0.03
113	60.17±0.67	50.01±0.26	47.65±0.59	45.45±0.23
114	55.50±0.34	55.40±0.50	54.96±0.25	51.14±0.92
116	85.35±0.71	84.37±0.46	81.61±0.76	74.69±0.32
119	62.20±0.09	55.64±0.16	30.49±0.05	27.71±0.03
120	44.91±0.13	44.46±0.16	43.92±0.20	43.88±0.13
121	63.52±0.14	60.85±0.15	59.66±0.06	54.93±0.45
122	68.03±0.07	66.55±0.36	65.45±0.15	62.89±0.29
125	23.67±0.06	21.99±0.05	21.76±0.09	18.57±0.06
126	65.59±0.20	64.62±0.14	64.45±0.14	61.77±0.20
127	64.25±0.28	62.72±0.14	59.94±0.21	55.24±0.02
128	58.42±0.22	57.64±0.20	56.62±0.02	55.53±0.01

129	66.80±0.33	65.43±0.27	43.99±0.66	43.91±0.06
131	69.30±0.02	68.54±0.04	67.87±0.03	65.78±0.03
132	26.09±0.09	24.17±0.10	21.70±0.07	17.67±0.08
134	48.27±0.16	49.08±0.59	44.67±0.32	39.72±0.09
135	69.81±0.14	69.21±0.17	65.62±0.08	61.77±0.23
TDM-2	77.70±0.09	73.43±0.03	72.74±0.06	63.65±0.04
TCM-3	89.19±0.08	84.92±0.01	79.70±0.07	71.79±0.09
LGG	67.40±0.02	55.40±0.10	53.96±0.06	53.17±0.06

Supplementary Figures

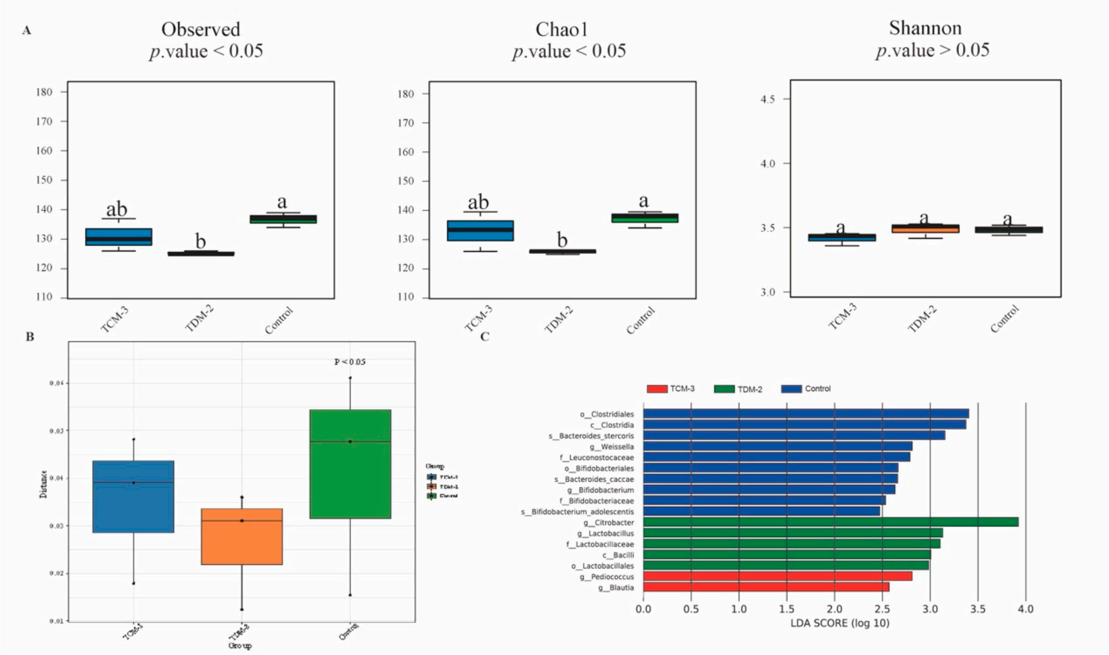

Figure S1. Analysis of Alpha diversity for bacterial communities. (A)Alpha diversity index of fecal microbiota in terms of microbial richness estimates Observed, Chao1 and diversity indices Shannon. (B) Comparison of fecal microbiota as affected by the TDM-2 and TCM-3 produced by linear discriminant analysis (LDA score > 2.5) generated by LEfSe analysis after incubation of the samples for 24 h. (C) Permutational multivariate analysis of variance (PERMANOVA) using distance matrices of 24h.

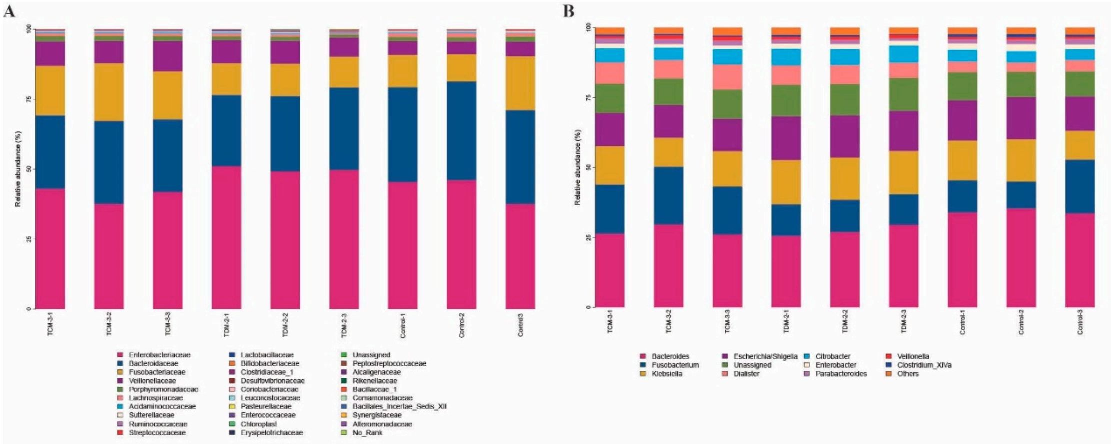

Figure S2. Effects of TDM-2 and TCM-3 supplementation on gut microbiota diversity. (A) Relative abundance of the gut microbiota at the genus levels after 24 h, respectively. (B) Relative abundance of the gut microbiota at the phylum level after 24 h, respectively.

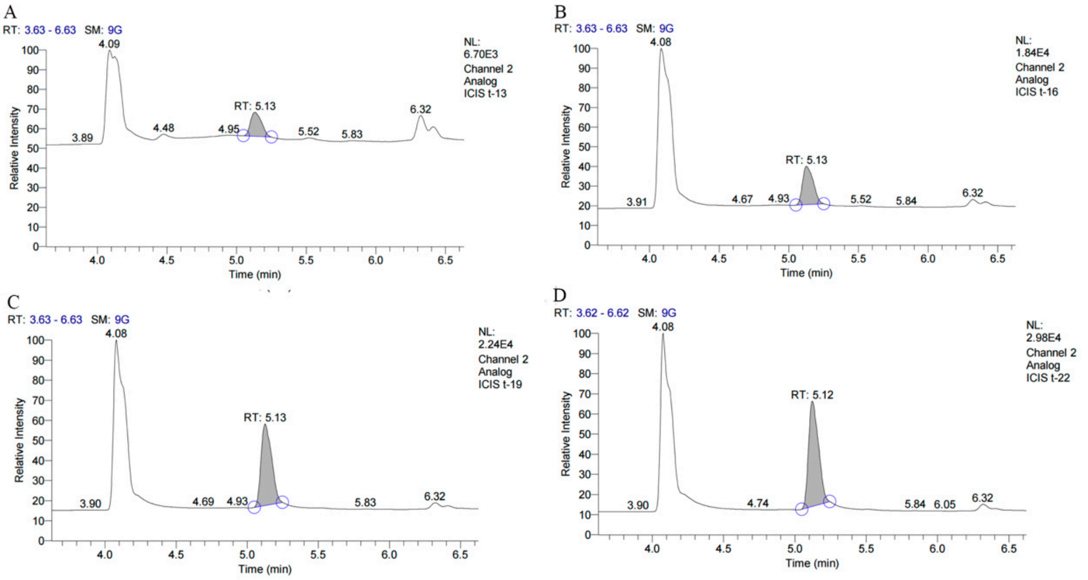

Figure S3. The GC-MS separation of fatty acid of *Lacticaseibacillus paracasei* TDM-2. (A) 0 h, (B) 6 h, (C) 12 h, (D) 24 h

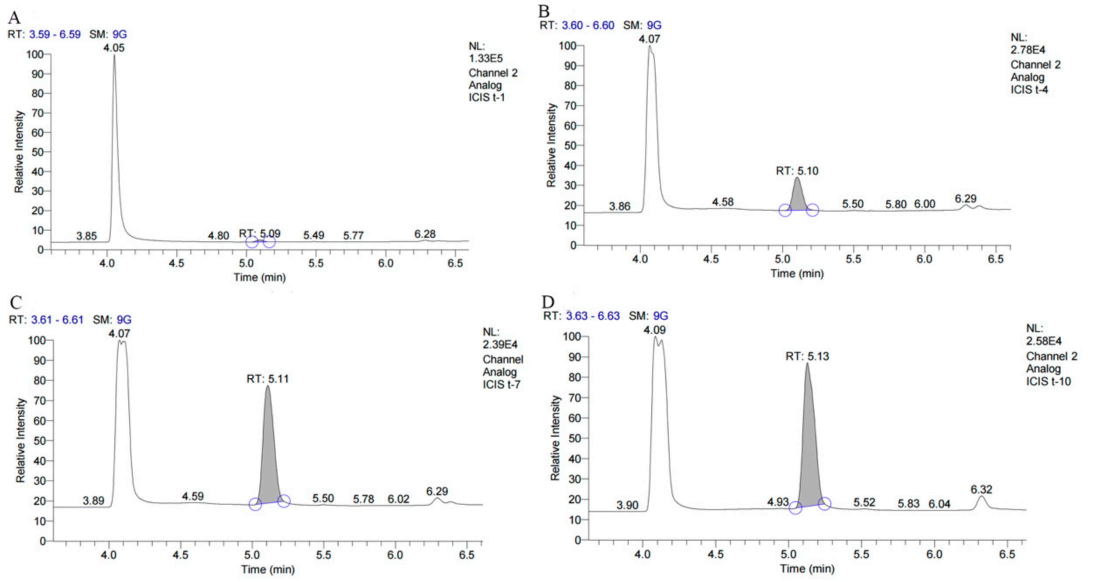

Figure S4. The GC-MS separation of fatty acid of *Pediococcus pentosaceus* TCM-3. (A) 0 h, (B) 6 h, (C) 12 h, (D) 24 h

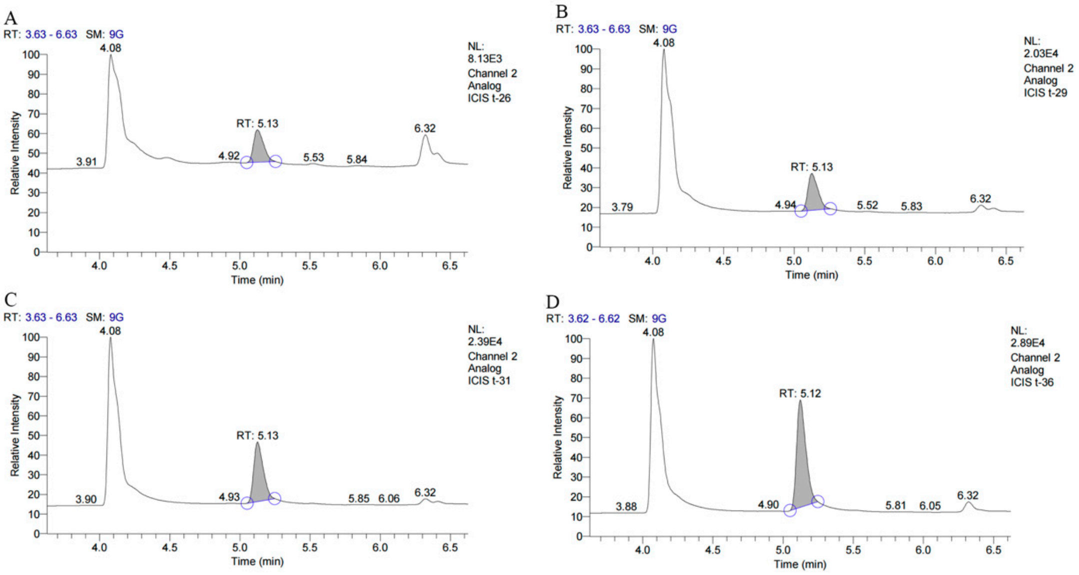

Figure S5. The GC-MS separation of fatty acid of control. (A) 0 h, (B) 6 h, (C) 12 h, (D) 24 h
